# Use of constitutive and inducible oncogene-containing iPSCs as surrogates for transgenic mice to study breast oncogenesis

**DOI:** 10.1186/s13287-021-02285-x

**Published:** 2021-05-27

**Authors:** Christine Nguyen, Julie P. T. Nguyen, Arnav P. Modi, Ihsaan Ahmad, Sarah C. Petrova, Stuart D. Ferrell, Sabrina R. Wilhelm, Yin Ye, Dorthe Schaue, Sanford H. Barsky

**Affiliations:** 1 Cancer Center and Institute for Personalized Medicine, California University of Science and Medicine (CUSM), 1501 Violet Street, Colton, CA 92324 USA; 2 Department of Radiation Oncology, David Geffen School of Medicine, University of California, Los Angeles, CA 90095-1714 USA

**Keywords:** Transgenic mice, Induced pluripotent stem cells (iPSCs), Breast oncogenesis, Differentiation, Constitutive and inducible models

## Abstract

**Background:**

Powerful constitutive and inducible transgenic / bitransgenic / tritransgenic murine models of breast cancer have been used over the past two decades to shed light on the molecular mechanisms by which the given transgenic oncogenes have interacted with other cellular genes and set in motion breast cancer initiation and progression. However, these transgenic models, as in vivo models only, are expensive and restrictive in the opportunities they provide to manipulate the experimental variables that would enable a better understanding of the molecular events related to initial transformation and the target cell being transformed.

**Methods:**

To overcome some of these limitations, we derived oncogene-containing induced pluripotent stem cell (iPSC) clones from tail vein fibroblasts of these transgenic mice and manipulated them both in vitro and in vivo in non-transgenic background mice. We created the iPSC clones with a relatively low M.O.I, producing retroviral integrations which averaged only 1 to 2 sites per retroviral plasmid construct used.

**Results:**

Most iPSC clones derived from each group displayed an essentially normal murine karyotype, strong expression of the exogenous reprogrammable genes and significant expression of characteristic endogenous murine surface stem cell markers including SSEA-1 (CD15), PECAM-1 (CD31), Ep-Cam (CD326), and Nectin (CD112), but no expression of their transgene. A majority (75%) of iPSC clones displayed a normal murine karyotype but 25% exhibited a genomically unstable karyotype. However, even these later clones exhibited stable and characteristic iPSC properties. When injected orthotopically, select iPSC clones, either constitutive or inducible, no longer expressed their exogenous pluripotency reprogramming factors but expressed their oncogenic transgene (PyVT or ErbB2) and participated in both breast ontogenesis and subsequent oncogenesis. When injected non-orthotopically or when differentiated in vitro along several different non-mammary lineages of differentiation, the iPSC clones failed to do so. Although many clones developed anticipated teratomas, select iPSC clones under the appropriate constitutive or inducible conditions exhibited both breast ontogenesis and oncogenesis through the same stages as exhibited by their transgenic murine parents and, as such, represent transgenic surrogates.

**Conclusions:**

The iPSC clones offer a number of advantages over transgenic mice including cost, the ability to manipulate and tag in vitro, and create an in vitro model of breast ontogeny and oncogenesis that can be used to gain additional insights into the differentiated status of the target cell which is susceptible to transformation. In addition, the use of these oncogene-containing iPSC clones can be used in chemopreventive studies of breast cancer.

**Supplementary Information:**

The online version contains supplementary material available at 10.1186/s13287-021-02285-x.

## Introduction

Powerful constitutive and inducible murine models of breast cancer have been used over the past decades to shed light on the molecular mechanisms by which transgenic oncogenes interact with other cellular genes and induce transformation. These transgenic models include single transgenics, bitransgenics, and tritransgenics. These models have afforded the opportunity to investigate the effects of the oncogenic transgene linked to a breast promoter like mouse mammary tumor virus (MMTV) on transformation. The bitransgenics afford to the opportunity to induce transformation via a turn on / turn off transgene mechanism. The tritransgenics may or may not include a turn on / turn off mechanism but afford a way to tag a target cell colorimetrically. Common models have included the FVB/N-Tg(MMTV-PyVT)634Mul/J; FVB-Tg(MMTV-ErbB2)NK1Mul/J; MMTV-erbB2/MMTV-cre/Rosa26LoxP; MMTV-PyVT/MMTV-cre/Rosa26LoxP; MMTV-rtT/tetO-erbB2; and MMTV-rtTA/tetO-PyVT among others [1–3]. However, these transgenic models are relatively expensive and restrictive in the opportunities they provide to gain a more specific understanding of the molecular events related to breast cancer initiation and progression. Specifically, these models do not allow for a precise determination of the target cell or its state of differentiation that is susceptible to its initial transformation. This is important because cancers are common diseases in people and yet, on a cellular level, are quite rare [4–6]. The vast majority of both sporadic spontaneous cancers and inherited germline cancers arise in single foci from singly transformed cells [7], despite the fact that, in the former, carcinogenic factors bathe fields of millions of potential target cells [6] and, in the latter, the predisposing germline mutations are present in every cell of a given organ and, in fact, every cell of the body [8, 9]. In the transgenic murine models of breast cancer, although the oncogenic transgene similarly is in every cell and although transgenic mice usually exhibit multifocal breast cancers, the vast majority of the cells within their inguinal mammary fat pads remain untransformed.

In the case of human breast cancer, the vast majority of spontaneous, sporadic breast cancers are solitary in nature. Human breast cancers which arise from the effects of exogenous estrogen from hormone replacement therapy are also solitary. Even in the setting of inherited germline mutated BRCA1 or BRCA2, which is present in all the cells of the breast, only solitary cancers or at most multifocal cancers limited to 2 or 3 foci arise [10–12]. Attempts to explain the rareness of breast cancer at a cellular level have invoked the multi-hit theory of carcinogenesis which basically opines that breast cancers do not occur unless there has been an accumulation of all of the necessary hits within the cell of origin [13, 14]. Although the multi-hit theory of breast carcinogenesis has also been invoked to explain such things as cancer latency which is the period between cancer initiation and emergence and the cancer-aging relationship where an accumulation of “hits” over a period of time are necessary for cancer emergence, the multi-hit theory falls short in explaining the rareness of transformation at a cellular level [15–20]. This is so because the germline inherited BRCA1/BRCA2 breast cancers are caused by only 1 or 2 hits and the spontaneous, sporadic breast cancers due to external radiation, hormone replacement therapy, dietary carcinogens, pesticides, etc. would be expected to bathe all the cells of the breast subjecting them to all of the “hits” required for carcinogenesis. Similarly, the transgenic murine models of breast cancer usually overexpress only a single oncogene, which again supports the hypothesis that breast cancer is not due to multiple hits.

Another hypothesis which has been invoked to explain the rareness of transformation has been the stem / progenitor cell compartmental theory of tumorigenesis [21–23]. That hypothesis opines that cancers including breast cancer contain a significant stem / progenitor cell compartment. The evidence for this belief is strong and multifaceted. For one, only the stem cell or progenitor cell subpopulation of a breast cancer is capable of self-renewal and multipotency. The proliferating subpopulation of a breast cancer is susceptible to radiation therapy and chemotherapy and other antiproliferative strategies, but breast cancer stem cells and progenitor cells resist such antiproliferative strategies. Breast stem and progenitor cancer cells express different stem cell-associated genes and pathways and have biomarkers of stemness that distinguish them from other tumor subpopulations. Breast cancer stem and progenitor cells are thought to be largely responsible for tumor relapses and recurrences in patients [21–23]. In transgenic models of breast cancer, subpopulations with stem/progenitor cell markers have also been identified. In both situations, although tumor stem / progenitor cells represent an important tumor cell subpopulation, their presence does not account for the rareness at a cellular level of the *initial* transformation.

In a recent observation, we advanced a different and novel hypothesis to explain the rareness of breast cancer at a cellular level despite the very high incidence of the disease in women. Our hypothesis was that transformation only occurs at a critical window of differentiation so that cells outside this critical window cannot transform**.** We reasoned that if we could obtain adult non-transformed tail vein fibroblasts from these transgenics and convert them to induced pluripotent stem cells (IPSCs) containing the oncogenic transgene, we might create a transgenic surrogate model of breast cancer that would not only be less expensive but would allow for a more precise study of both differentiation and transformation both in vitro and in vivo.

## Results

### Generation of IPSC clones

**Fig. 1:**
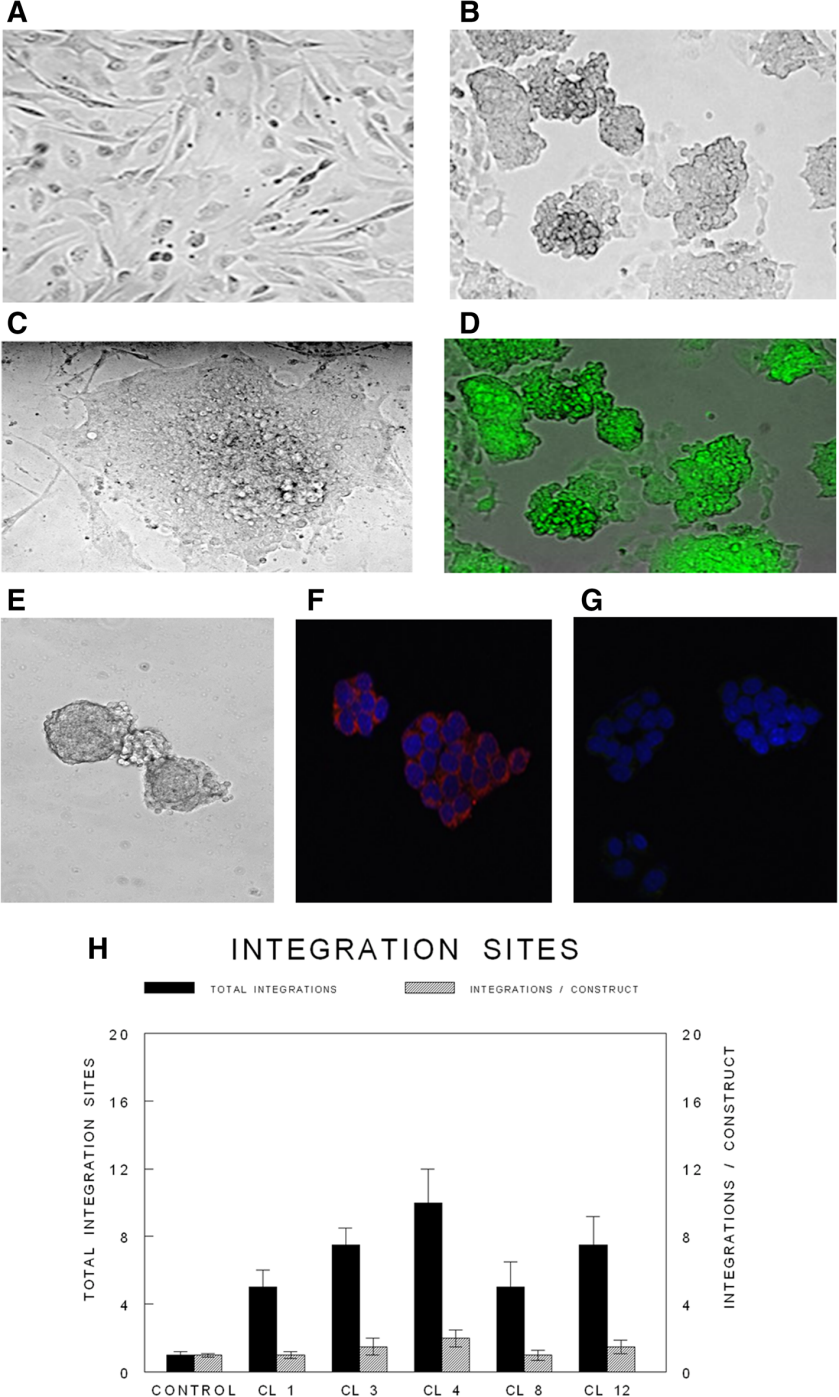
Retroviral transfection of tail vein fibroblasts and selection of iPSC clones. Different transgenic, bitransgenic, tritransgenic, and background mice were used to isolate and prepare tail vein fibroblasts. All tail vein-derived fibroblasts appeared similarly (**a**). pMXs-mSox2, pMXs-mOct3/4, pMXs-mKlf4, pMXs-mc-Myc, and pMX-GFP were separately transfected into a Platinum-A retroviral packaging cell line to produce retroviruses. The retroviruses from the preceding step were equally mixed and transduced into fibroblasts. Colonies became visible approximately 8–15 days after the retroviral infection. Morphology of an emerging colony of the monolayer by phase contrast (**b**), its appearance on a feeder layer (**c**), and its GFP autofluorescence (**d**) are displayed. A emerging embryoid body from one of the iPSC clones is also depicted (**e**). Most emerging iPSC clones were positive for alkaline phosphatase, a known iPSC marker. Select iPSC clones exhibited typical markers of pluripotent stem cells, e.g., SSEA-1 (**f**) with control (**g**). The number of retroviral integration sites in representative clones ranged from an average of 1.0 to 2.0 / retroviral construct based on qPCR

We used the following transgenics, bitransgenics, and tritransgenics to generate iPSC clones: the FVB/N-Tg(MMTV-PyVT)634Mul/J; the FVB-Tg(MMTV-Erbb2)NK1Mul/J; the MMTV-cre/Rosa26LoxP; the MMTV-erbb2/MMTV-cre/Rosa26LoxP; the MMTV-PyVT/MMTV-cre/Rosa26LoxP; the MMTV-rtT/tetO-erbb2; and the MMTV-rtTA/tetO-PyVT. We obtained tail vein fibroblasts from each of these groups. We also obtained fibroblasts from non-carrier control FVB background mice. The tail vein fibroblasts (Fig. 1a) were transfected with a cocktail of stem cell-inducing and reporter genes which were generated through a retroviral packaging cell line. Five plasmids with their respective inserts from Cell Biolabs Inc. were used to produce retroviruses: pMXs-mSox2, pMXs-mOct3/4, pMXs-mKlf4, pMXs-mc-Myc and pMX-GFP. All these vectors were separately transfected into the Platinum-A Retroviral Packaging Cell Line, Amphotropic, to produce retroviruses expressing their respective stem cell or reporter gene. The retroviruses obtained were equally mixed and transduced into the tail vein fibroblasts to induce iPSCs according to established methods.

After two cycles of retroviral transduction, the transduced fibroblasts were cultured in embryonic stem (ES) medium (DMEM containing 15% FBS (vol/vol), 2 mM L-GIn, 1 × 10^− 4^ M nonessential amino acids, 1 × 10^− 4^ M 2-mercaptoethanol, 10 ng/ml LIF, and 50 mg/ml of penicillin and streptomycin). Each day, the fibroblasts were fed fresh ES medium to generate iPSC clones. Approximately 20–30 clones emerged from each of the transduced fibroblast groups (Fig. 1b). Select single iPSC clones showing a characteristic 3D morphology (Fig. 1c) and expressing GFP fluorescence (Fig. 1d) were selected and cultured in 24-well plates containing a SNL feeder layer. Embryoid bodies emerged (Fig. 1e–g). The MOI used in the creation of our iPSC clones was 5.0 for each of the 5 retroviral plasmid vectors used, allowing for two rounds of retroviral transfection. We calculated the number of retroviral integrations in 4 representative iPSC clones which illustrated a range of 1.0 to 2.0 insertions per retroviral plasmid vector used (Fig. 1h). We conducted qPCR using primers derived from the 5′ LTR end of the retroviral construct using as a control, tail vein fibroblasts retrovirally transfected with a single retroviral plasmid GFP construct. With clonal dilution of the MOI in the control, we were able to select single fibroblast clones that expressed GFP from only a single integration site. The number of retroviral integrations ranged from 1.0 to 2.0 /retroviral plasmid in our iPSC clones and therefore was not very high and certainly not high enough to likely trigger insertional mutagenesis. Furthermore, none of the iPSC clones appeared transformed either through the loss of contact inhibition or uncontrolled cell division.

### Identification, selection, and characterization of iPSC clones

Alkaline phosphatase, a known embryonal stem cell marker, was used initially to confirm the identity of the iPSC clones. The vast majority of the clones derived from the groups of tail vein fibroblasts were indeed alkaline phosphatase positive. After single clones were obtained, we used immunofluorescence to confirm the expression of known IPSC markers, e.g., Sox2, nestin, and undifferentiated ES cell surface antigen, SSEA-1 (mouse-specific stage-specific embryonic antigen-1) (Fig. 1f,g).

**Fig. 2:**
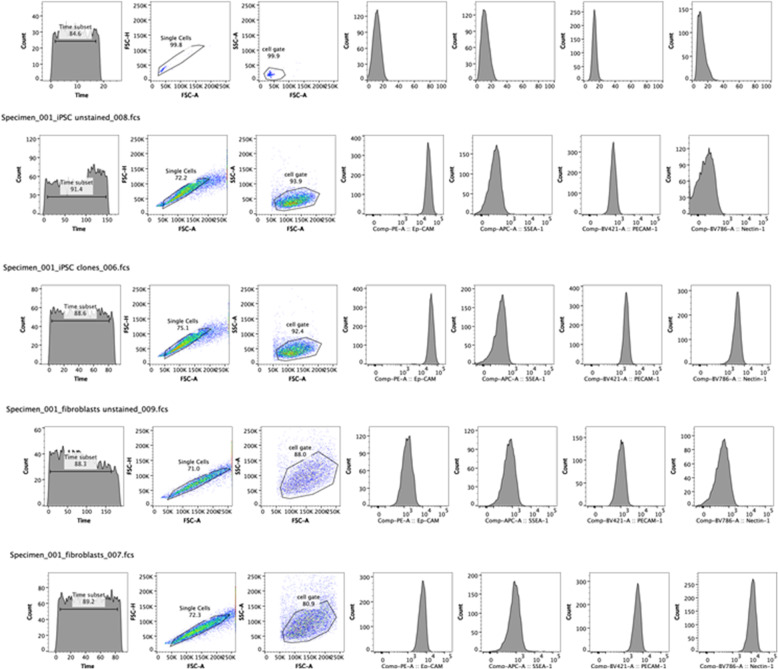
Flow cytometry of iPSC endogenous surface markers. Flow cytometry of representative iPSC clones shows the strong multi-channel expression of endogenous surface iPSC markers including SSEA-1, Ep-Cam (CD326), PECAM-1 (CD31), and Nectin (CD112) with the latter two markers expressed more strongly. Additional iPSC clones were analyzed (Supplement 1)

Multi-channel flow cytometric analysis using murine SSEA-1 (CD15), PECAM-1 (CD31), Ep-Cam (CD326), and Nectin (CD112) was used to further characterize our iPSC clones. All of these iPSC surface markers were expressed in the representative iPSC clones examined, but Nectin and PECAM were more strongly expressed and there was some heterogeneity in expression among the different iPSC clones examined. Two of the iPSC clones examined also expressed GFP but some clones did not. The surface IPSC markers we chose were specifically not the reprogramming gene products we used for retroviral transfection but rather endogenous gene products which were known to characterize murine iPSCs. The flow cytometry results are depicted (Fig. 2, Supplement 1). Interestingly some of these surface markers were also expressed in non-iPSC clones such as murine fibroblasts.

We also conducted karyotype studies of select iPSC clones. Approximately 75% of the clones studied expressed a completely normal female murine karyotype of 40 chromosomes but 25% of the iPSC clones examined displayed a genomically unstable karyotype with lost chromosomes, extrachromosomes, and rare translocations (Supplement 2). Despite evidence of genomic instability, these later clones expressed iPSC surface markers, all 4 reprogrammable genes, pluripotency, and retroviral integrations in the range of 1.0–2.0 / clone.

**Fig. 3:**
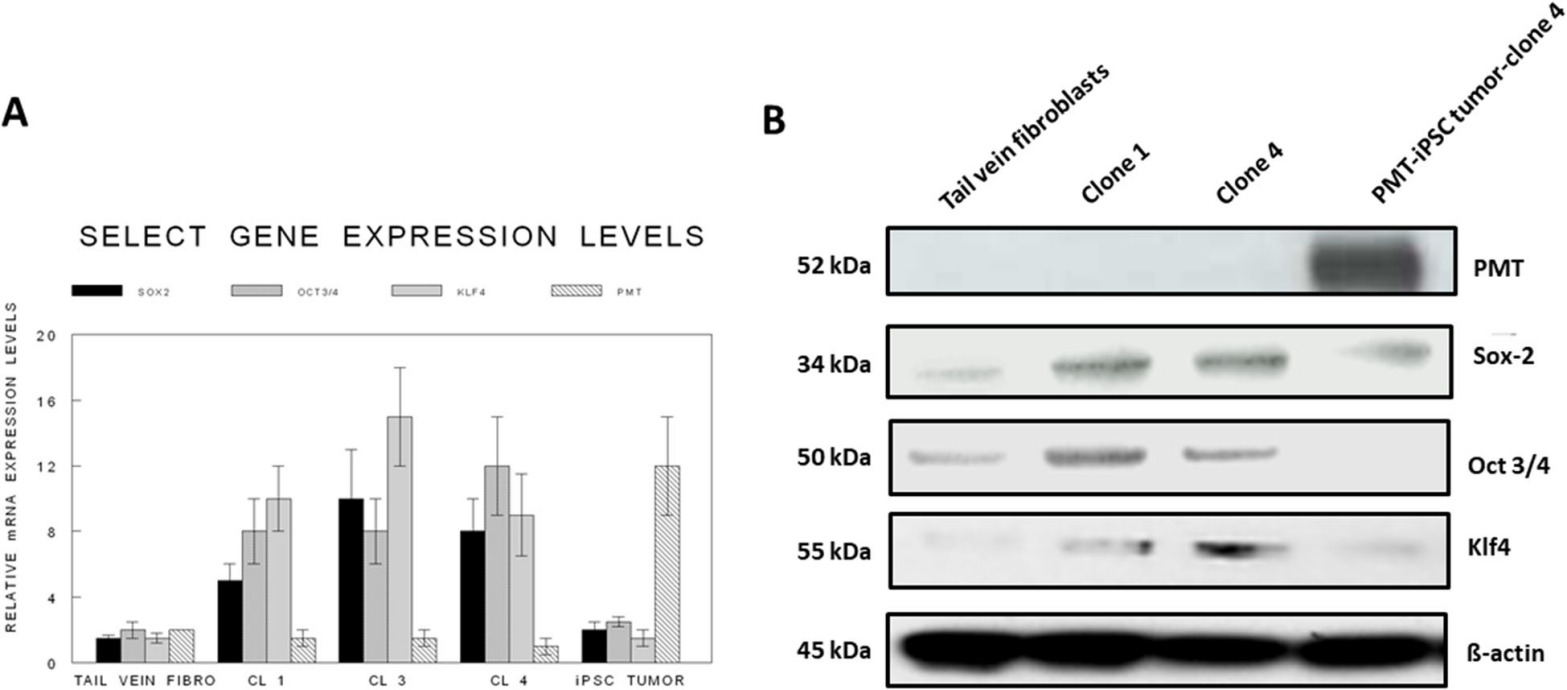
Select gene expression levels in the different groups by RT-qPCR and Western blot analysis. Strong expression of the iPSC reprogramming genes was in evidence in representative clones but not in the tail vein fibroblasts. During in vivo oncogenesis, the expression of the exogenous pluripotency reprogramming factors no longer occurred but were replaced by strong expression of the oncogenic transgene, e.g., PyVT (PMT) by RT-qPCR (**a**). The same observations were confirmed by Western blot (**b**)

Strong expression of the iPSC reprogramming genes was also in evidence in both representative karyotypically stable and unstable iPSC clones but not in the tail vein fibroblasts by both RT-qPCR (Fig. 3a) and Western blot (Fig. 3b). During in vivo oncogenesis, the expression of the exogenous pluripotency reprogramming factors no longer occurred but were replaced by strong expression of the oncogenic transgene, e.g., PyVT (PMT) by RT-qPCR and Western blot (Fig. 3a,b).

The iPSC clones obtained from each of the groups of tail vein fibroblasts grew similarly and expressed identical IPSC markers overall but there was considerable heterogeneity within each group in terms of morphology, expression of stem cell markers, doubling time, and expression of the respective transgene. Genotyping revealed the presence of the respective transgenes, PyVT, and mutated erbB2 in nearly all the iPSC clones derived from the transgenics but predictably not from the non-carrier FVB background mice (data not shown). Clones that expressed all of the expected iPSC markers and grew with typical iPSC morphology were selected for subsequent in vivo studies. The selected iPSC clones when injected orthotopically gave rise to mammary cancers which strongly resembled those arising spontaneously within their transgenic parents. The iPSC clones selected for in vivo implantation did not express either transgene in vitro by either RT-qPCR (Fig. 3a) or Western blot (Fig. 3b). Nor was the expression of either transgene within these clones induced by dexamethasone in vitro (data not shown).

### Pluripotent differentiation of iPSC clones

**Fig. 4:**
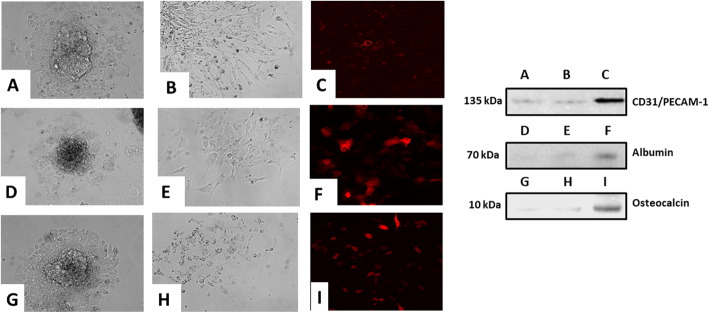
Pluripotent differentiation of iPSC clones in vitro. Differentiation of iPSCs into various lineages at different time points is depicted (left). Undifferentiated iPSC clones were induced to differentiate into endothelial cells, depicted at day 7 (**a**) and then at day 15 (**b**). Similarly, undifferentiated iPSC clones were induced to differentiate into hepatic cells, depicted at day 7 (**d**) and at day 15 (**e**). Additionally, undifferentiated iPSC clones were induced to differentiate into osteocytes, depicted at day 7 (**g**) and at day 14 (**h**). Each of the lineage differentiations were confirmed by representative marker studies which included CD31 (endothelial) (**c**), albumin (hepatic) (**f**), and osteocalcin (**i**). Western blot from the lineage-specific differentiated cells confirmed their respective endothelial, hepatic, and osteocyte differentiation (right)

Select iPSC clones were differentiated in vitro into endothelial (Fig. 4a–c), hepatic (Fig. 4d–f), and osteogenic (Fig. 4g–i) lineages according to established methods [24–26]. Their morphology was monitored over 14 days. They first developed the morphology of embryoid bodies that continued to differentiate along the designated lineage. At the end of the differentiation period, each lineage revealed by immunofluorescence studies lineage-specific biomarkers which included CD31 (endothelial) (Fig. 4c), albumin (hepatic) (Fig. 4f), and osteocalcin (Fig. 4i). Western blot for the appropriate lineage-specific marker confirmed the immunofluorescence data (Fig. 4a–i).

### Animal studies

#### Implantation studies

FVB female background was used to inject the selected iPSC and control clones. Clones were injected into the cleared inguinal mammary fat pads and into non-orthotopic subcutaneous sites. Mice were monitored over the next 2–4 months for mammary gland ontogenesis and oncogenesis.

#### Histological studies

Emerging tumors were studied by routine light microscopy. The breast cancers arising in the PyVT transgenics strongly resembled the breast cancers arising from the PyVT-iPSC clones injected into the mammary fat pad. Occasionally, both non-carrier iPSC clones when injected orthotopically and PyVT-iPSC clones when injected non-orthotopically gave rise to teratomas (data not shown). The breast cancers arising in the ErbB2 transgenics strongly resembled the breast cancers arising from the mammary fat pad-injected ErbB2-iPSC clones. Occasionally, ErbB2-iPSC clones when injected non-orthotopically also gave rise to teratomas (data not shown).

#### Fluorescence and immunocytochemical studies

**Fig. 5:**
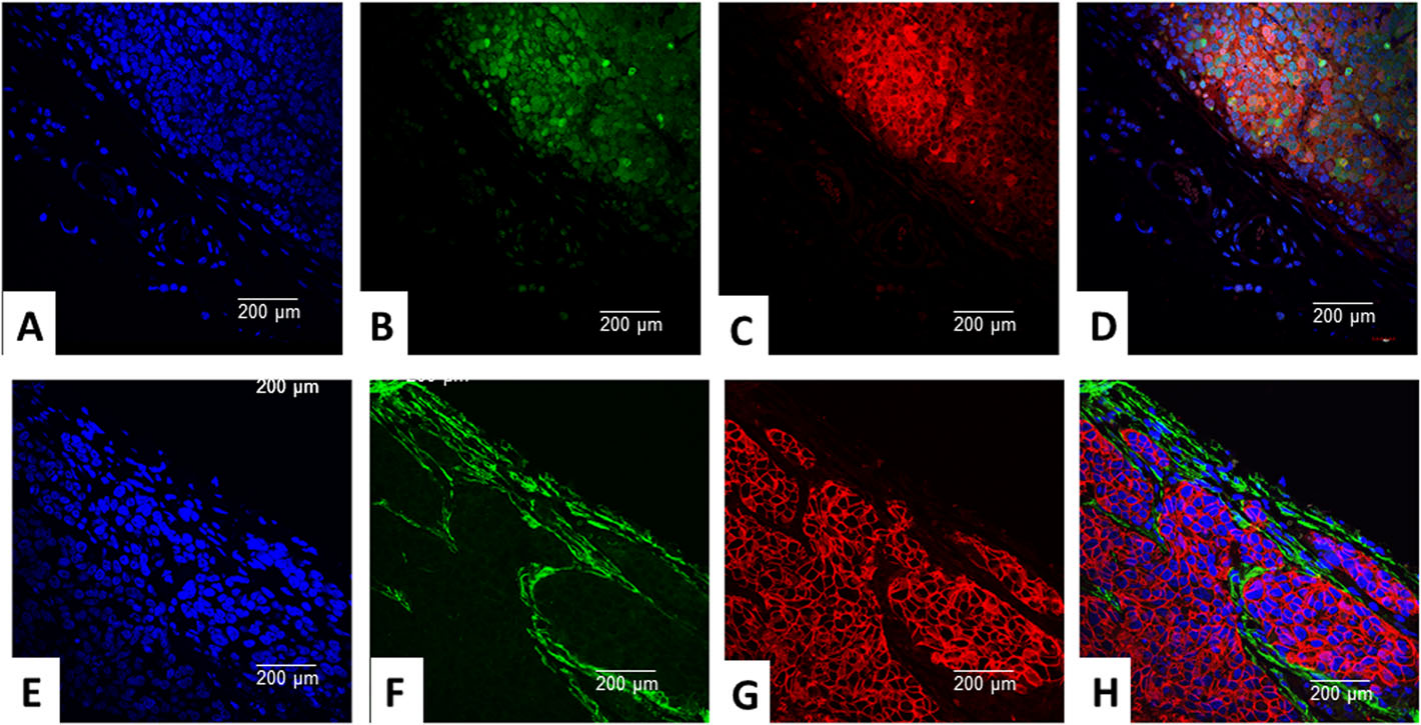
Trifluorescence studies of extirpated tumors in the transgenic IPSC clones. Triple fluorescence studies on PyVT-iPSC generated extirpated tumors demonstrated DAPI blue nuclear autofluorescence (**a**), GFP green autofluorescence (**b**), and Alexa Fluor® 594 red immunofluorescence using goat anti-rat added to rat monoclonal to PyVT antigen (**c**) and its merged overlay (**d**). Strong cytoplasmic expression of the PyVT antigen is observed. Similarly, triple fluorescence studies on ErbB2-iPSC generated extirpated tumors demonstrated DAPI blue nuclear autofluorescence (**e**), GFP green autofluorescence (**f**), and Alexa Fluor® 594 red immunofluorescence using goat anti-rabbit added to rabbit polyclonal antibody to ErbB2 (**g**) and its merged overlay (**h**). Strong membrane expression of the ErbB2 antigen is observed

The iPSCs derived from the following transgenics FVB/N-Tg(MMTV-PyVT)634Mul/J and FVB-Tg(MMTV-erbb2)NK1Mul/J, when injected into the cleared inguinal mammary fat pads of background FVB mice, after approximately 60 days and 120 days respectfully, developed tumors. PyVT-iPSC clones gave rise to mammary carcinomas which exhibited dual GFP autofluorescence and PyVT cytoplasmic red immunofluorescence (Fig. 5a–d). ErbB2-iPSC clones also gave rise to mammary carcinomas which exhibited dual GFP autofluorescence and ErbB2 membrane red immunofluorescence (Fig. 5e–h). However, in a number of areas, GFP expression decreased while ErbB2 expression increased (Fig. 5h). This dichotomy of expression was not initially appreciated but was not without a possible explanation. The GFP construct was retrovirally transduced into our clones whereas the Her-2/neu construct represented the original transgene present in the tail vein fibroblasts and linked to a breast-specific promoter. The genes used in retroviral reprogramming including the retroviral reporter gene (GFP) would be expected to be only transiently expressed as the iPSC clones differentiate whereas the genes linked to the breast-specific promoter would be expected to be more strongly expressed as breast ontogenesis and breast oncogenesis progress.

**Fig. 6:**
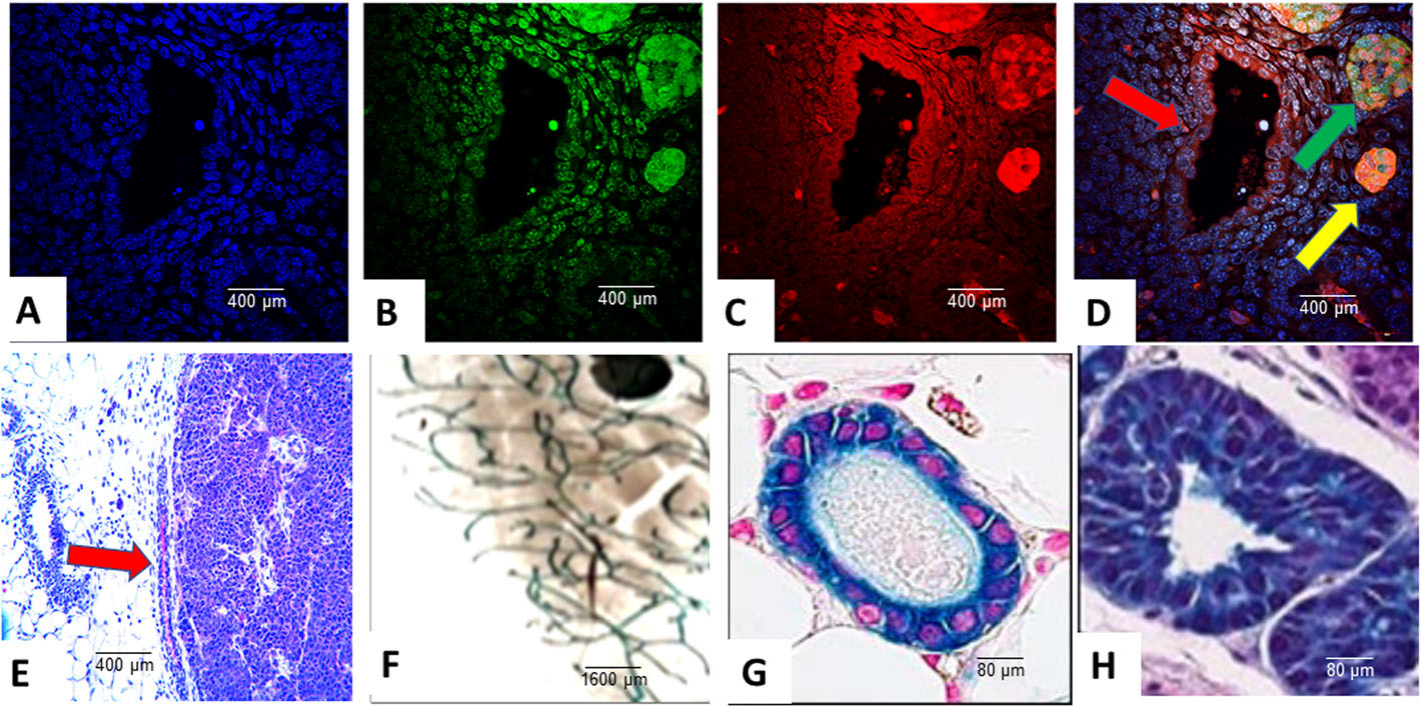
Trifluorescence and colorimetric studies of stages of oncogenesis on select iPSC clones. Triple fluorescence studies on PyVT-iPSC clone-derived extirpated tumors displayed DAPI blue nuclear autofluorescence (**a**), GFP green autofluorescence (**b**), and Alexa Fluor® 594 red immunofluorescence using goat anti-rat added to rat monoclonal to PyVT antigen (**c**) and its merged overlay (**d**). Expression of PyVT is detected not only within the areas of invasive carcinoma (yellow arrow) but also within the normal breast ducts (red arrow) and breast ducts containing ductal carcinoma in situ (DCIS) (green arrow) whereas murine angiogenesis (dark areas) did not exhibit any GFP autofluorescence or immunofluorescence. Colorimetric immunocytochemistry studies utilizing A Fast Red precipitating chromogenic substrate system coupled with alkaline phosphatase-conjugated goat anti-rat and rat anti-PyVT revealed red chromogenicity not only within the invasive carcinoma but also within adjacent normal ducts (red arrow) (**e**). The iPSC clones derived from the tritransgenic MMTV-PyVT/MMTV-cre/Rosa26LoxP, when injected orthotopically, gave rise to normal ductal system development (**f**,**g**) and stages of breast cancer progression (**f**,**h**), both identified colorimetrically. The iPSC clones were capable of behaving orthotopically no different than that which was observed in their tritransgenic parent

More detailed analyses of the extirpated PyVT-iPSC tumors (Fig. 6a–d) revealed dual GFP autofluorescence and PyVT cytoplasmic red immunofluorescence not only within the areas of invasive carcinoma (yellow arrow) but also within the normal breast ducts (red arrow) and breast ducts containing ductal carcinoma in situ (DCIS) (green arrow) whereas angiogenesis (dark areas) did not exhibit any GFP autofluorescence or immunofluorescence. The injected PyVT-iPSC clones did not therefore differentiate into endothelial cells that resulted in angiogenesis. Angiogenesis occurred from murine precursor cells and not from the injected iPSCs. The same observations were made with injected iPS-ErbB2 cells (data not shown).

A Fast Red precipitating chromogenic substrate system couple with alkaline phosphatase-conjugated goat anti-rat and rat anti-PyVT revealed red chromogenicity not only within the invasive carcinoma and DCIS areas but also within adjacent ducts (red arrow) (Fig. 6e). Similar results were observed with the ErbB2-iPSC tumors (data not shown).

In the iPSC clones derived from the tritransgenic MMTV-PyVT/MMTV-cre/Rosa26LoxP, when injected orthotopically, gave rise to both breast ontogenesis as well as breast oncogenesis (Fig. 6f–h), both areas of which expressed a colorimetric marker observed in the original transgenic. The iPSC clones were capable of behaving orthotopically no different than that which was observed in their tritransgenic parent. The iPSC clones which were derived from the tail vein fibroblasts did not express the colorimetric tag even when their tritransgenics developed the tag within their mammary fat pads. Similarly, in the MMTV-rtT/tetO-erbB2 and the MMTV-rtTA/tetO-PyVT bitransgenics, the derived iPSCs did not express their transgene even when their bitransgenic parents were given doxycycline prior to tail vein harvesting.

#### RT-qPCR and Western blot studies

**Fig. 7:**
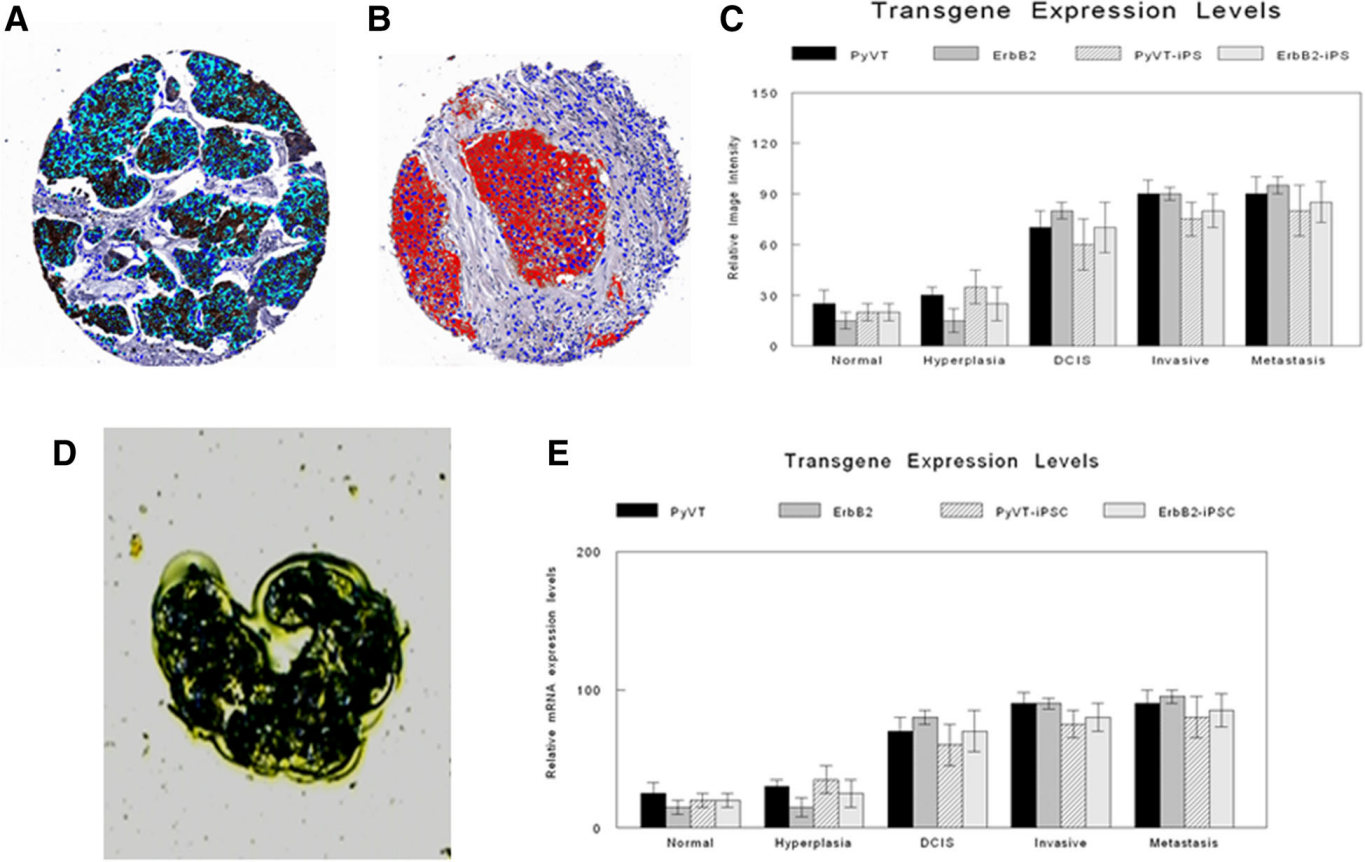
Digital image analysis with specific recognition algorithms (SRAs) and quantitation of transgene expression. Digital image analysis of relative fluorescence and colorimetric immunocytochemistry of representative TMA cores illustrate quantitative PyVT cytoplasmic immunoreactivity (**a**) and quantitative ErbB2 membrane immunoreactivity (**b**). Relative immunocytochemical intensity levels of both transgenes in normal ducts, ducts with hyperplasia, ducts with DCIS, and invasive carcinoma and metastatic carcinoma in the tumors arising in the original transgenics *v* the tumors arising from the injected iPSC clones are depicted (**c**). Laser-capture microdissection (**d**) of tumoral areas subjected to RNA extraction and real-time RT-PCR depicts relative mRNA expression levels in the same groups and tumoral areas (**e**)

Specific studies were carried out to demonstrate and quantitate expression of the oncogenic transgenes, PyVT and ErbB2, in the various cell lines, iPSC clones, and emerging tumors (Figs. 3a, b and 7a–e). These studies included RT-PCR (Fig. 3a), Western blot (Fig. 3b), and laser-capture microdissection-based (Fig. 7d) RT-qPCR (Fig. 7e). Only the transgenic mammary cancers (and their metastases) and the mammary cancers (and their metastases) arising from transgene-containing iPSC clones injected orthotopically expressed the relevant transgene (Figs. 3a, b and 7c, e).

#### Quantitative digital image analysis

Digital image analysis was performed on virtual microscopic scanned images from a tissue microarray (TMA) created from the mammary fat pads of the different iPSC transgene-injected groups. Using previously developed cytoplasmic and membrane recognition algorithms [27, 28], respectively for the PyVT (cytoplasmic signals) (Fig. 7a) and the ErbB2 (membrane signals) (Fig. 7b), the study quantitated both the fluorescent and the immunocytochemical signals of the respective transgenes in normal ducts, ducts with hyperplasia, ducts with carcinoma in situ (DCIS), and invasive carcinoma and compared the relative intensities. There was a progressive increase in transgene signal in normal, hyperplasia, ductal carcinoma in situ (DCIS), and invasive / metastatic cancer with both transgenes (Fig. 7c). This pattern was similarly observed both within the iPSC derived tumors as well as within the tumors arising spontaneously within the parental transgenics.

### Overall studies

**Fig. 8:**
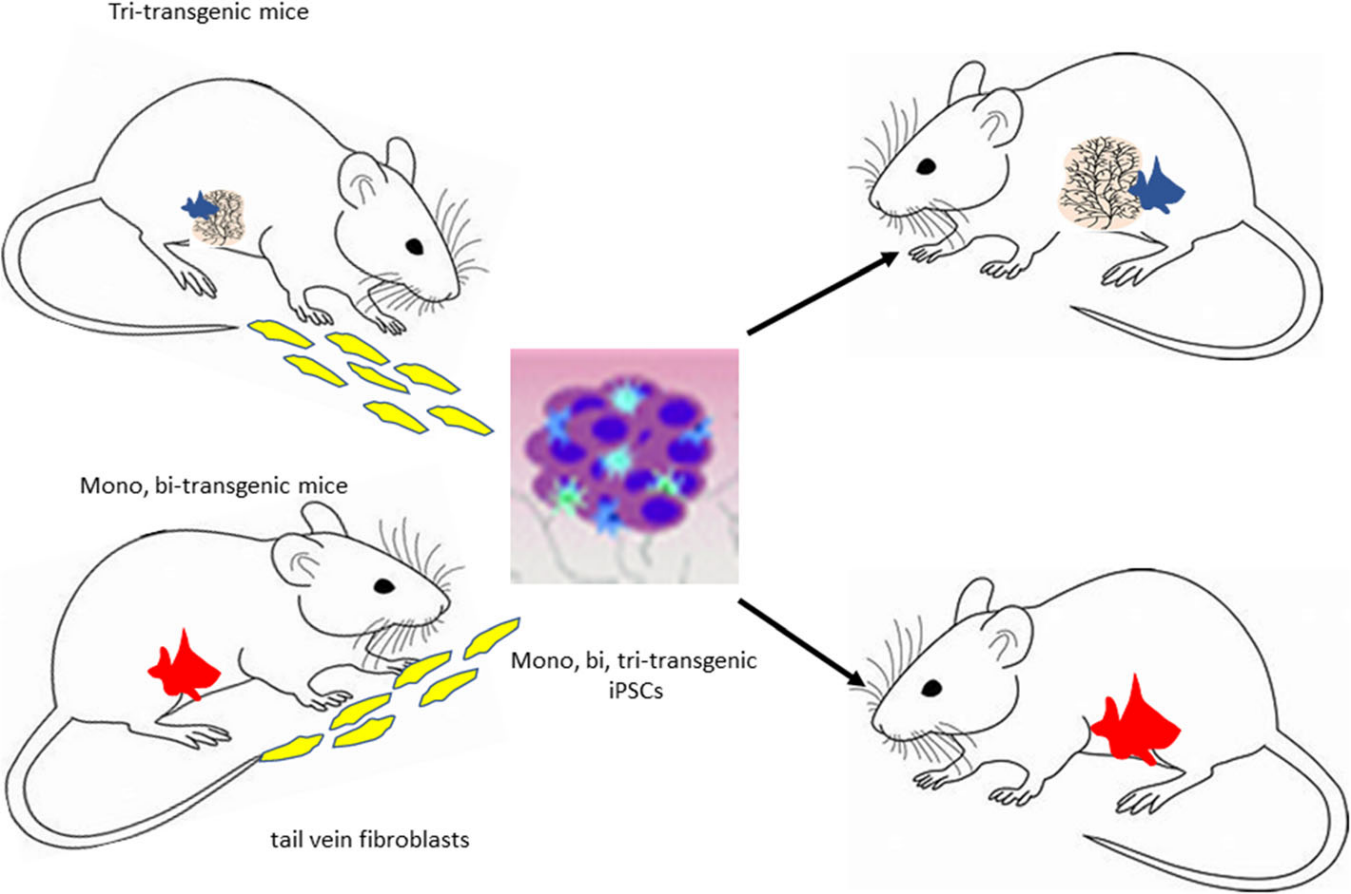
Oncogene-containing iPSCs as surrogates for transgenic mice. Schematic depicts the use and efficacy of using these iPSC clones to duplicate both breast ontogenesis and breast oncogenesis occurring in the transgenics but only when injected orthotopically. These iPSC clones not only represent a transgenic surrogate model of breast cancer but one that allows for a more precise study of both differentiation and transformation

Based on all of the aforementioned studies, the oncogene-containing iPSC clones and their transformed progeny represent transgenic surrogates that can allow for greater insights into breast oncogenesis (Fig. 8).

## Discussion

Transgenic models of breast cancer provide powerful models to study breast oncogenesis [1–3]. Using these models, the actions of a number of breast oncogenes and the molecules they interact with have been elucidated. Interestingly, the breast cancers which emerge in most of these models go through the precancerous stages of breast cancer progression before they progress to invasive and metastatic breast cancer. Although many of these models produce multifocal breast cancers, sometimes termed inaccurately polyclonal cancers, the mice harboring the cancers show a normal ductal-alveolar system in the adjacent normal breast indicating that most of the epithelial cells comprising the ductal-lobular system of the breast do not transform and therefore mimic, at least in part, the human situation in both spontaneous, sporadic as well as inherited germline breast cancers where only limited foci give rise to cancer.

Although the transgenic, bitransgenic, and tritransgenic models of breast cancer are powerful models, they are expensive. A given transgenic costs 5–10× the cost of a background mouse and this cost difference would rise 10^2^–10^3^ fold in the bitransgenic and tritransgenic plus the costs of maintaining the breeding colonies. Surrogate iPSC clones derived from transgenics, bitransgenics, and tritransgenics grown in background mice save the majority of these costs. We also reasoned that if we could obtain adult non-transformed tail vein fibroblasts from these transgenics and convert them to induced pluripotent stem cells (IPSCs) containing the oncogenic transgene, we might create a transgenic surrogate model of breast cancer that would not only be less expensive but would allow for a more precise study of both differentiation and transformation both in vitro and in vivo and specifically test our hypothesis of whether the initial transformation is related to a critical window of differentiation.

Our findings indicate that this approach works in that the derived iPSC clones from every transgenic, bitransgenic, and tritransgenic model revert back to an IPSC state in vitro despite the presence of an oncogenic transgene and (in the case of the bitransgenic and tritransgenic), modifying inducers and/or markers in which the transgene and the modifying inducers and/or markers are not expressed. When implanted orthotopically in background mice, the oncogene-containing iPSC clones exhibit the same biology as its transgenic, bitransgenic, and tritransgenic parent. Select iPSC clones from each group when injected orthotopically exhibited their constitutive and inducible transgene expression and initiated both breast ontogenesis and subsequent multistage oncogenesis just like their transgenic parents. The iPSC clones therefore represent a true transgenic surrogate. In addition, our iPSC clones can be perturbated in vitro and manipulated in vivo by implanting them non-orthotopically as well. Since the iPSC clones can be labeled or tagged during their in vitro creation, their fate can be monitored in vivo and they can be retrieved from their in vivo environment at any stage of breast ontogenesis or oncogenesis and studied further in vitro.

It should be emphasized that only a minority of clones in each oncogene-containing iPSC set gave rise to breast ontogenesis and breast oncogenesis. The majority of clones from each iPSC set gave rise to teratomas and this was especially so when the clones were injected non-orthotopically. The whole process of iPSC and oncogene-containing iPSC is a bit stochastic. Not all clones are created equal. Although the majority of iPSC clones exhibited a normal murine karyotype, a minority did not and showed genomic instability. This instability has been observed previously in retrovirally mediated integrating reprogramming methods [29]. Interestingly, however, both types of iPSC clones exhibited a characteristic iPSC phenotype with identifying iPSC surface markers, all 4 reprogrammable genes, pluripotency, and retroviral integrations in the range of 1.0–2.0 / clone.

But in our genesis of many clones, we have the luxury of selecting those clones that differentiate along the pathways we wish to study, and in this setting, it is the pathways of breast ontogenesis and oncogenesis. Given clones that, in fact, did this, consistently did so.

As one example of the advantage provided by the iPSC clones, we manipulated their differentiation state both in vitro and in vivo to eliminate the critical window of differentiation that allows for both breast ontogenesis and breast oncogenesis. Our findings indicated that both in vitro and in vivo differentiation regulate breast oncogenesis. If we drive the transgenic iPSC clones to differentiate into endothelial, hepatic, or osteogenic lineage directions, not only is the transgene not expressed but the differentiated clones will not differentiate in vivo into breast nor breast cancer when injected into the mammary fat pad. When we inject the undifferentiated iPSC clones into the mammary fat pad, they first presumably differentiate into mammary stem cells, then into the breast ductal system and then select cells which transform into precancerous and invasive cancerous epithelium. The transgene becomes transformative only when it can act on the iPSCs that have begun to differentiate along a mammary lineage. When the select undifferentiated iPSC clones are injected into a non-orthotopic site, however, they do not differentiate into a mammary gland nor do they participate in mammary oncogenesis. This observation means two things. Paracrine factors in the mammary fat pad microenvironment which we do not totally understand are necessary to induce differentiation of the iPSCs into mammary stem cells capable of further differentiation into the mammary ductal-alveolar system and that only after this has happened, can transformation occur. Possible transcriptional mechanisms involved in this process have been studied previously [30–33]. At non-orthotopic sites, this induction does not occur. Only when this differentiation occurs but not before, can breast oncogenesis be initiated. A critical window of differentiation must exist before breast oncogenesis can commence. The transgene drives this oncogenesis and its expression increases with the progressive stages of breast cancer progression.

The iPSC clones derived from the tritransgenic MMTV-PyVT/MMTV-cre/Rosa26LoxP, when injected orthotopically, gave rise to both breast ontogenesis and breast oncogenesis, both areas of which expressed a colorimetric β-galactosidase marker observed in the original tritransgenic. The iPSC clones were capable of behaving orthotopically no different than that which was observed in their tritransgenic parent. The iPSC clones which were derived from the tail vein fibroblasts did not express the colorimetric tag even when their tritransgenics developed the tag within their mammary fat pads. Similarly, in the MMTV-rtT/tetO-erbB2 and the MMTV-rtTA/tetO-PyVT bi-transgenics, the derived iPSCs did not express their transgene even when their bitransgenic parents were given doxycycline prior to tail vein harvesting. This is because the MMTV promoter does not drive the related gene expression in the tail vein nor in its derived iPSC clones and therefore they are primed to express their inducible elements only in the same location as their bitransgenic or tritransgenic parents. The many different iPSC clones we have created from tail vein fibroblasts of the transgenics, bitransgenics, and tritransgenics enumerated above which include doxycycline-inducible bitransgenics will allow us to treat the various iPSC clones in vitro with differentiating agents, examine their morphogenesis in vitro, inject them into cleared fat pads, observe the degree of mammary gland morphogenesis and/or tumorigenesis in whole mounts, retrieve the cells along a time course, sort the cells for known markers of lineage and differentiation, analyze the retrieved cells for gene expression patterns, and study them further in vitro. All of the iPSC clones have GFP as reporter; some of the iPSC clones, when injected into the cleared mammary pad, may express a second conditional Rosa26 reporter (β-galactosidase). For example, since the MMTV-cre/Rosa26LoxP bitransgenic exhibits blue histochemical staining (with X-gal) of the entire mammary ductal tree and the ductal-lobular units when the MMTV promoter is stimulated, it would be expected that the mammary fat pad when injected with tail vein-derived iPSC clones from the bitransgenics would do the same. Since the MMTV-erbb2/MMTV-cre/Rosa26LoxP or MMTV-PyVT/MMTV-cre/Rosa26LoxP exhibits both blue histochemical staining of the ductal tree and the breast cancer grossly and microscopically when the MMTV promoter is stimulated, it would be anticipated that the injected tail vein-derived iPSC clones from either tritransgenic would do the same and this is exactly what was observed. We believe these different combinations of iPSC clones provide a powerful surrogate experimental system to study both mammary morphogenesis as well as mammary tumorigenesis so that we can specifically examine the critical window of differentiation hypothesis.

Although other investigators have demonstrated that a single murine mammary stem cell, when injected into the cleared mammary fat pad, is sufficient to generate an entire mammary ductal tree [34, 35] and that murine embryonic stem cells, when induced to undergo hematopoietic differentiation in vitro and then injected into the mammary fat pad, are able to exhibit mammary morphogenesis [36], we believe that our study is the first to observe both mammary ontogenesis as well as oncogenesis.

Our iPSC transgene model further allows for both in vitro and in vivo dissection of those factors that may more precisely define the critical window of differentiation. Using the many different iPSC clones that we have created from the oncogenic transgenics, we can experiment with a various number of differentiating agents attempting to induce a mammary lineage differentiation, something that has not been done previously from oncogene-containing iPSC clones derived from adult fibroblasts. To date and to the best of our knowledge, no one has successfully driven iPSCs or oncogene-containing iPSC clones derived from adult fibroblasts into mammary gland differentiation in vitro, though both iPSCs and embryonic stem cells, using their derived embryoid bodies (EBs), have been differentiated into hepatocyte, hematopoietic, osteogenic, and endothelial lineages in vitro using cocktails of defined cytokines and growth factors [37–41]. Murine embryonic stem cells (mES), but not iPSCs, did differentiate in 3D Matrigel chambers into ductal-alveolar structures that expressed ductal epithelial and myoepithelial markers. However, they were negative for secretory markers of β-casein and whey acidic protein (WAP). More recent studies have successfully induced mammary gland differentiation in vitro but by only first overcoming lineage-specific restrictions on mammary differentiation in iPSC cells derived from adult mesenchymal sources by either co-culture experiments or using non-neural ectoderm or epithelial cells rather than adult fibroblasts as starting material [42–44]. None of these studies have used oncogene-containing IPSC clones derived from any source to induce either mammary ontogenesis or oncogenesis. As mentioned previously, mES, when differentiated in vitro and then injected, exhibited mammary morphogenesis [36]. Obviously, the mammary microenvironment in both situations plays a key role in mammary morphogenesis. Still, from the mammary epithelial perspective, regulatory networks orchestrated by key transcription factors (TFs) also play a role in mammary differentiation. In mES, a set of core TFs, notably Oct4, Sox2, and Nanog, form an autoregulatory network and act cooperatively to activate genes capable of maintaining the embryonic (ESC) state and, at the same time, silence the expression of genes involved in lineage-specific differentiation [30, 32, 45]. It has been shown that Slug and Sox9 also act cooperatively to regulate the mammary stem cell state [31]. If we can more precisely differentiate our transgene-iPSC clones into mammary gland differentiation in vitro, then we can derive sorted subpopulations of differentiating cells to determine at what point in vivo ontogenesis and oncogenesis is enhanced or lost. We could then select the injected iPSC clones that show the most robust mammary gland morphogenesis and that exhibit differentiation along all three mammary gland lineages: luminal cells, secretory cells, and myoepithelial cells in vitro to use our model in a reverse direction.

Those iPSC clones that show the most promise in terms of mammary gland development can then be harvested from the fat pads by collagenase, dispase, and trypsin digestion over a time course to monitor lineage-specific differentiation. We could gate and sort the retrieved cells based initially on GFP. According to the epithelial differentiation hierarchy model [41], mouse mammary glands consist of a hierarchy of several cell types which include multipotent stem cells which are able to regenerate entire mammary glands in mice at the single cell level, bipotent progenitor, unipotent progenitor, and differentiated cells, which are delineated by different combinations of cell surface markers. So we could use these cell surface markers and fluorescence-activated cell sorting (FACS) to analyze the cells and divide them into the following fractions: multipotent stem cells (CD29hiCD49hi CD24+ESA−), luminal progenitor (CD61+CD49loESA+CD24+CD29lo) and ductal cells (CD24+CD61−CD29lo), as well as parity-induced mouse mammary epithelial cells (PI-MECs) (CD24hiCD49lo).

Some of these cells, e.g., luminal progenitor cells, have been thought to be the targets of erbB2 gene-induced tumorigenesis [46, 47]. Other investigators have challenged these observations, arguing that multipotent PI-MECs are the true targets of ErbB2 tumorigenesis [48]. Certainly, more insights are needed using our iPSC transgenic model. One basic question we hope to answer is the time course of oncogene expression. PyVT or ErbB2 expression did not occur in the derived iPSC clones in vitro nor was it induced by dexamethasone despite the fact that at least theoretically a dexamethasone responsive MMTV promoter lies upstream of the transgene. What this means is that there are other in vivo mammary factors required to stimulate MMTV-transgene expression.

Oncogene-induced transformation requires not only the expression of the oncogene but also the activation of oncogene-mediated pathways. Therefore, our transgene-iPSC model should allow us to investigate in which subpopulation(s) and at what time point(s) the oncogene-mediated pathway(s) are activated. The two oncogenes, PyVT and EerbB2, activate very similar pathways involving cellular kinases and phosphatases [49–52], recruitment of activated c-Src, activation of Ras/Erk, and PI3K/Akt signaling [2]. Integration of these multiple pathways ultimately induces cellular transformation. Therefore, it is reasonable to hypothesize that during the period before the critical window of differentiation has been opened and after the critical window has been closed that the pathways activated by the oncogene are neutralized by events related to the early and late stages of differentiation. Therefore, we could study the gene expression patterns of the oncogene-activated pathways in the unsorted and sorted subpopulations over the time course of mammary ontogenesis and oncogenesis. Another basic question in mammary oncogenesis derived from the transgene-containing iPSC clones is the expression of those genes responsible for iPSC induction, e.g., Sox2, Oct3/4, Klf4, and Myc. Certainly one would expect that the expression of these genes might decrease with mammary ontogenesis and oncogenesis. As far as mammary oncogenesis is concerned, it has also been shown that ErbB2 and PyVT may regulate cancer stem cells and cancer stem cell pathways [49–52]. Therefore, a study of the expression patterns of the four key genes used for iPSC induction, more global transcriptome profiling, and additional microarray analysis on these same cellular subpopulations might shed insight into the critical window of differentiation as well as other critical steps of breast cancer initiation and promotion. Using oncogene-containing iPSC clones and in the case of the bitransgenics and tritransgenics, oncogene-containing iPSC clones containing modifying inducers and/or markers allows us to approach the issues with a greater ability to perturbate the experimental variables.

The ability to perturbate our iPSC clones in vitro with differentiating agents including agents which promote mammary differentiation may allow us to screen for chemopreventive agents that extinguish the critical differentiation window of transformation. Although other investigators have derived iPSC clones from many different sources including transgenic mice, we believe that, to our knowledge, this is the first study to specifically derive iPSC clones from breast oncogenic mice where the derived iPSC clones specifically silence the oncogenic transgene expression until re-injected into the mammary fat pad where the oncogenic transgene is re-expressed. This model allows insight into the process of both breast ontogenesis as well as breast oncogenesis.

## Conclusions

In summary, although transgenic models of breast cancer provide powerful models to study breast oncogenesis, we reasoned that if we could obtain adult non-transformed tail vein fibroblasts from these transgenics and convert them to induced pluripotent stem cells (IPSCs) containing the oncogenic transgene, we might create a transgenic surrogate model of breast cancer that would not only be less expensive but would allow for a more precise study of both differentiation and transformation both in vitro and in vivo. The iPSC clones indeed offer a number of advantages over transgenic mice including cost, the ability to manipulate and tag in vitro, and create an in vitro model of breast ontogeny and oncogenesis.

## Experimental procedures

### Generation of IPSC clones

Tail vein fibroblasts were isolated as discussed under “Animal studies” and prepared for retroviral transfection. A Platinum-A Retroviral Packaging Cell Line, Amphotropic (Cell Biolabs, San Diego, CA) was prepared in the following manner. After quickly thawing the cells in a 37 °C water bath, the thawed cell suspension was transferred into a 15-mL tube containing 10 mL of culture medium. This was centrifuged for 5 min at 1300 to 1500 rpm. The supernatant was discarded, and the cell pellet was disrupted by finger tapping. Two milliliters of culture medium was added to the tube, and the cell suspension was gently pipetted. The cell suspension was transferred to a 10-cm culture dish containing 8 ml of culture medium. The culture plate with the cells was incubated at 37 °C and 5% CO_2_. A Murine Stem Cell Factor Retroviral Vector Set (4 Genes) with pMX-GFP reporter Retroviral Vector (Cell Biolabs) was obtained and each plasmid was separately transfected according to the manufacturer’s instructions with vectors: pMX-GFP, pMXs-mOct3/4, pMXs-mSox2, pMXs-mc-Myc, and pMXs-mKlf4 [53]. For each type of transduction, 2.0 × 10^5^ tail vein fibroblasts were plated in a 60-mm culture dish in complete culture medium (DMEM with high glucose, 10% FBS, 1% PS), washed, and transduced dropwise with a mixture of vector-containing supernatant filtered through a .45-μm cellulose acetate syringe filter (Whatman, Tisch Scientific, North Bend, OH). The process was repeated.

### Identification and selection of iPSC clones

After two cycles of retroviral transduction with an MOI of 5.0, the transduced fibroblasts were cultured in embryonic stem cell (ES) medium (DMEM containing 15% FBS (vol/vol), 2 mM L-GIn, 1 × 10^− 4^ M nonessential amino acids, 1 × 10^− 4^ M 2-mercaptoethanol, 10 ng/ml LIF, and 50 mg/ml of penicillin and streptomycin). Each day, the fibroblasts were fed with fresh ES medium to generate iPS clones. Colonies became visible approximately 8 days after the retroviral infection in transduced fibroblasts from each of the groups. The morphology of many of the iPSC clones was similar and typical of iPSC clones. However, some of the clones appeared more fibroblastic and more rapidly growing. Around 15 days after retroviral transduction, representative iPSC clones were picked and cultured in 24-well plates seeded with SNL feeder cells [54].

#### Alkaline phosphate staining

The culture medium was aspirated, and cells washed twice with 2 ml of PBS. The cells were fixed with 0.5 ml of 4% paraformaldehyde in PBS for 1–2 min. Two milliliters of fix solution was added and incubated at room temperature for 1 to 2 min. One milliliter of freshly prepared AP staining solution was then added. The cells were incubated in the dark (wrapped with foil or in a dark container) at room temperature for 10 to 20 min. The color change was closely monitored and stopped when the color turned bright to avoid non-specific staining. The reaction was stopped by aspirating the AP staining solution and washing the wells twice with 2 ml of 1× PBS. The cells were then coverslipped with mounting medium to prevent drying. AP expression resulted in a red or purple stain, while the absence of AP expression resulted in no stain. The plate was then stored at 4 °C.

#### iPSC marker immunofluorescence

The iPSC clones were confirmed as IPSCs with a battery of rabbit anti-mouse IPSC markers including Oct3/4, Sox2, c-Myc, mKlf4, nestin, and SSEA-1 (Thermo Fisher Scientific, Waltham, MA) The secondary antibody was an Alexa Fluor 594-conjugated goat anti-rabbit (Thermo Fisher Scientific), all used with the manufacturer’s conditions. In order to immobilize the iPSC clones, glass-bottom dishes were coated with Cell-TEK adhesive. The adherent iPSC cells were then fixed with 4% paraformaldehyde, after permeabilizing with TX-100 and blocking with normal goat serum. The iPSC clones were then incubated with the primary antibodies, washed, and followed by the secondary antibodies. The dishes were finally mounted with Vectorshield mounting medium with DAPI (#H-1200) (Vector Laboratories, Burlingame, CA) and viewed with an Olympus Fluoview-1000 confocal scanning system under different wave lengths.

#### Karyotype analysis

Standard karyotypic analysis was conducted with colchicine-induced, metaphase analysis of 40 spreads, Giemsa staining, and G-band analysis with imaging software (Creative Biolabs, Shirley, NY) (Supplement 2).

#### Flow cytometry

Select iPSC clones were studied with multi-channel flow cytometry using known antibodies to endogenous cell surface determinants present on iPSC clones: PE anti-mouse Ep-CAM (clone G8.8); APC anti-mouse SSEA-1 (clone MC480); BV421 anti-mouse PECAM-1 (clone390); cat# 5633; BV786 anti-mouse Nectin-2 (clone 829,038); (All from Becton Dickinson, Franklin Lakes, NJ). We used a 3-laser, 14-color LSRFortessa™ (BD Biosciences, San Jose, CA), and analyzed 10,000–20,000 cells using FlowJo (FlowJo, LLC, Ashland, OR) (Supplement 1).

#### qPCR, RT-qPCR, and Western blot studies

The number of retroviral integration sites in select iPSC clones was studied with qPCR. The primers used were derived from the MuLv 5′LTR. The primer pair consisted of as follows: forward primer: 5′-GTGCCCCAAGGACCTGAAAT-3′; reverse primer: 5′-GGAACAGCGAGACCACAAGT-3′. Control consisted of retrovirally transfected fibroblasts with GFP and limiting dilution MOI to exhibit only a single integration site.

RT-qPCR was used to study relative mRNA expression levels of the exogenous reprogramming genes in select iPSC clones used to create the iPSCs from fibroblasts. The genes and primers used were as follows: Klf4: forward primer: 5′-CAAGTCCCCTCTCTCCATTATCAAGAG-3′; reverse primer: 5′-CCACTACGTGGGATTTAAAAGTGCCTC-3′, Oct3/4: forward primer: 5′-CACGAGTGGAAAGCAACTCA-3′; reverse primer: 5′-AGATGGTGGTCTGGCTGAAC-3′; Sox2: forward primer: 5-ACATGTGAGGGCTGGACTGCGAAC-3′ and reverse primer: 5′-GAAGCGCCTAACGTACCACTAGAAC-3′. Primers used for PyVT were as follows: forward primer: 5′-TTTGGAACACCAACCCGAGA-3′; reverse primer: 5′-ATCCAGGTCCAGCCAGTCTAT-3′; primers used for ErbB2 were as follows: forward primer: 5′-ATTGGCTCTGATTCACCGCA-3′; reverse primer: 5′-CAAGCCCTCGAGACCACAAT-3′. The primers used for the analysis of β-actin were as follows: forward primer: 5′-GGCACCCAGCACAATGAAG-3′; reverse primer: 5′-GCCGATCCACACGGAGTACT-3′. An additional housekeeping control was used, GAPDH. The primers used for the analysis of GAPDH were as follows: forward primer: 5′-ATGGGGAAGGTGAAGGTCG-3′; reverse primer: 5′-GGGGTCATTGATGGCAACAATA-3′.

These studies were used to investigate the iPSC clones for expression of their relevant exogenous reprogrammable genes and relevant oncogenic transgenes. They were also used to evaluate the same within the emerging murine tumors as discussed in the section on “Animal studies.” Freshly picked iPSC clones and clones which had been frozen and quickly thawed as well as extirpated tumors described under “Animal studies” were made available for study. RNA quality and quantity were determined by measuring absorbance at 260 and 280 nm. Oligo(dT) primers (Integrated DNA Technologies, Coralville, IA) were used with Superscript II reverse transcriptase (Invitrogen, Waltham, MA) for cDNA synthesis from 1 μg total RNA extracted from the transgenic iPSC clones and the non-carrier iPSC clones. qPCR and RT-qPCR reactions were run on a 7500 Real-Time PCR system (Applied Biosystems, Inc., Foster City, CA). Gene expression was detected with SYBR Green Master Mix. Relative gene expression was determined by normalizing to β-actin using the Δ C _T_ method with 7500 System SDS software (Applied Biosystems, Foster City, CA).

For western blot studies, fresh and / or frozen material was quick thawed and made available for study. Cells and extirpated tumors were extracted with buffer (1% Triton X-100, 150 mM NaCl, 10 mM sodium phosphate, 10 mM EDTA). The samples were then centrifuged at 13,000*g* at 4 °C for 15 min. Protein concentrations were determined using the BCA protein assay (Pierce Biotechnology, Rockford, IL). Samples containing equal protein were boiled in Laemmli buffer under reducing conditions, run on a 4–15% SDS-polyacrylamide gel, and transferred to a PVDF membrane that was then incubated with the respective primary antibodies which included rabbit anti-mSOX2, anti-mOct3/4; anti-mKlf4 (all from Abcam, Cambridge, MA); rat anti-PyVT (Santa Cruz Biotechnology, Santa Cruz, CA) and rabbit anti-ErbB2 (Thermo Fisher Scientific); goat anti-mCD31/PECAM-1; anti-mosteocalcin and anti-malbumin (R&D Systems, Mineapolis, MN) and HRP-labeled goat anti-rat, goat anti-rabbit IgG or HRP-conjugated Ant-goat IgG secondary antibody (Cell Signaling Technology, Danvers, MA). Bound antibodies were detected by a chemiluminescent detection system (West Femto) (Pierce Biotechnology) according to the manufacturer’s instructions or with the Supersignal West Dura extended Duration Substrate (Pierce Biotechnology). A monoclonal antibody to glyceraldehyde 3-phosphate dehydrogenase (GAPDH) (Santa Cruz Biotechnology) was used to normalize for protein loading. A CCD Imaging system, the ChemiDoc™ MP Imaging System (Bio Rad, Hercules, CA), was used to quantitate the signal.

### Pluripotent differentiation of iPSC clones

#### Differentiation studies

Pluripotent differentiation was carried out along three different lineages; endothelial, hepatic, and osteogenic. Each lineage differentiation strategy involved two steps, formation of embryoid bodies (EB) and then subsequent lineage differentiation. As the differentiation process was occurring, the cells in each group were monitored by phase contrast microscopy.

For endothelial differentiation, the formation of embryoid bodies (days 0–6) occurred as follows: the iPSC clones which were growing on feeder layers were dispersed by treatment with trypsin-EDTA and collected by centrifugation at 800 rpm for 3 min. Cells were then resuspended in Knockout DMEM (Thermo Fisher Scientific) supplemented with 15% knockout serum replacement (KSR), 1% nonessential amino acids (MP Biomedicals, Santa Ana, CA), 1% 2-mercaptoethanol, 1% penicillin/streptomycin, and 1% l-glutamic acid. The iPSC clones were then transferred to ultra-low attachment six-well plates and cultured free floating at a density of 2 × 10^5^ cells/2 ml/well to induce the formation of embryoid bodies (EB). Half of the medium was replaced daily. After EB formation for 5 days, EBs were seeded on gelatin-coated six-well culture plates and cultured in DMEM medium with 10% FBS, 50 ng/mL hVEGF, 100 ng/mL hFGF-b, 10 ng/mL hIL-6, 2 U/mL hEPO and 50 U/ml penicillin, and 50 mg/ml streptomycin. The medium was changed every 2 days. The differentiation program was finished at day 16.

For hepatic differentiation, formation of embryoid bodies occurred under identical conditions as for endothelial differentiation. At day 8, the medium was replaced by knockout DMEM supplemented with 10% KSR, 1% nonessential amino acids, 1% l-glutamic acid, 1% DMSO, and 100 ng/ml hepatocyte growth factor (HGF) to induce hepatocyte-like cells. Half of the medium was replaced daily. The differentiation program was finished at day 16.

For osteogenic differentiation, formation of embryoid bodies occurred under identical conditions as for endothelial differentiation. After EB formation for 5 days, EBs were seeded on gelatin-coated six-well culture plates and were started culturing in osteogenic medium. The osteogenic medium was DMEM supplemented with 10% FBS, 10 nM dexamethasone, 50 mg/L ascorbic acid, 10 mM β-glycerophosphate, 50 U/ml penicillin, and 50 mg/ml streptomycin. The medium was changed every 3 or 4 days. The differentiation program was finished at day 16.

#### Biomarker studies

For each of the lineages, proof of successful differentiation was obtained by the detection of specific biomarkers for each lineage: CD31 for endothelial, albumin for hepatic, and osteocalcin for osteogenic. Cultures of the differentiated cells which grew as monolayers were subjected to double immunofluorescent studies using the following combinations of antibodies: rabbit anti-mouse CD31, rabbit anti-mouse albumin, rabbit anti-mouse osteocalcin followed by Alexa Fluor 594-conjugated goat anti-rabbit (all antibodies from Abcam, Cambridge, MA) The adherent monolayers were then fixed with 4% paraformaldehyde, after permeabilizing with TX-100 and blocking with normal goat serum. The spheroids were then incubated with the respective primary antibodies according to the manufacturer’s specifications, followed by washing with PBS 4–5 times and then followed by the secondary antibody again according to the manufacturer’s specifications. The dishes were finally mounted with Vectorshield mounting medium with DAPI (#H-1200) (Vector Laboratories) and viewed with an Olympus Fluoview-1000 confocal scanning system. Confirmatory studies using the same lineage-specific antibodies were carried out by Western blot.

### Animal studies

All transgenic, bitransgenic, and tritransgenic mice used were either purchased (The Jackson Laboratory Biomedical Research Institute, Bar Harbor, Maine) or bred. These included FVB/N-Tg(MMTV-PyVT)634Mul/J; FVB-Tg(MMTV-ErbB2)NK1Mul/J;MMTV-cre/Rosa26LoxP; MMTV-erbB2/MMTV-cre/Rosa26LoxP; MMTV-PyVT/MMTV-cre/Rosa26LoxP; MMTV-rtT/tetO-erbB2; and MMTV-rtTA/tetO-PyVT). Each group consisted of 10 4-week-old females. In addition, 100 non-carrier (FVB background mice) were obtained from the same vendor to serve as both controls and recipients of the implanted iPSC clones.

#### Tail vein procurement studies

Tail vein fibroblasts from all groups were isolated by cutting 5 cm of tail from 2-month-old mice, peeling the dermis and mincing the tail tips into 1-cm pieces. A pair of pieces was plated in a 600-mm collagen I-coated dish (BD Biosciences, Bedford, MA) with 5 ml DMEM containing 10% FBS (Sigma-Aldrich, St. Louis, MO). After 5 days of incubation, fibroblasts migrated out of the tail pieces and were transferred to new dishes and allowed to proliferate.

#### Implantation and harvesting studies

Clones were injected into the cleared inguinal mammary fat pads of the background FVB mice. The mammary fat pads were previously cleared at 3–4 weeks of age according to accepted procedures [55]. To test the tumorigenesis of the iPSC clones, iPSC clone derived from each of the transgenic groups as well as non-carrier controls were implanted. The iPSC clones obtained from each group that were subjected to in vitro differentiation were also similarly injected. iPSC clones of all the groups were also implanted non-orthotopically in areas such as the flank and back. Approximately, 5 × 10^5^ cells were used for each injection. The mice were observed for the next 4–8 weeks. In some mice, emerging tumors were obvious. In any case after this period of time, the mammary fat pad and the non-orthotopic sites were extirpated. Extirpated areas were either snap frozen or immediately processed for RT-PCR, Western blot, and real-time RT-PCR analysis and routine light microscopy, trifocal immunofluorescence, and digital image analysis. Mammary fat pads containing mammary carcinomas arising within the original transgenics, bitransgenics, and tritransgenics were similarly extirpated and processed.

#### Laser-capture microdissection studies

For laser-capture microdissection studies and subsequent RT real-time PCR, frozen sections (8 μ) were obtained from the mammary fat pads, fixed in 70% ethanol, stained with hematoxylin, and progressively dehydrated in 70%, 95%, and 100% ethanol, followed by xylene and air drying. Other frozen sections were stained with hematoxylin directly without fixation. Selected areas which included areas of normal ducts, ducts with hyperplasia, ducts with DCIS, and areas of invasive carcinomas and pulmonary metastasis were microdissected using a Pixcell II Laser Capture Microdissection 788 Laboratory System (Arcturus, Inc., Mountain View, CA) and stored in microdissection caps (CapSure Macro LCM Caps, Arcturus, Inc.) with RNA*later* (Thermo Fischer Scientific, Inc.) at − 80 °C.

#### RT-qPCR and Western blot studies

These studies have been enumerated previously under the section “Identification and selection of iPSC clones.”

#### Histological studies

Fresh-frozen and paraffin-embedded tissues of the extirpated tissues including tumors were processed according to standard protocols involving dehydration, paraffin embedding, sectioning, and staining with hematoxylin and eosin as well as cutting sections that were left unstained.

#### Colorimetric studies with β-gal

X-gal (5-Bromo-4-chloro-3-indolyl b-D-galactoside) was purchased (Sigma-Aldrich). Both fresh-frozen tissues cut on a cryostat and fixed and dehydrated tissues embedded in paraffin were processed. Slides were washed in LacZ Wash Buffer (PBS containing 0.01% sodium deoxycholic acid, 0.02% Nonidet-P40, 2 mmol/1MgC1_2_) three times for 5 min and subsequently incubated in X-gal staining solution (1 mg/rnlS-Bromo-4-chloro-3-indolyl S-O-Galactoside, 2 mmol/1 MgC1.2, 5 mmol/1 potassium ferrocyanide, 5 mmol/1 potassium) or alternatively a commercially available β-gal staining set was used according to the manufacturer’s instructions (Roche Diagnostics Corp., Indianapolis, IN). After 3 washes in PBS, the samples were postfixed in 0.2% glutaraldehyde (Electron Microscopy Sciences) washed in distilled water, counterstained with Nuclear Fast Red (NFR, Biomeda Foster City, CA), dehydrated in graded alcohols, cleared in xylene, and coverslipped.

#### Fluorescence and immunocytochemical studies

Sections of the extirpated tissues and tumors were then treated by target antigen retrieval solution (DAKO, Carpinteria, CA) in a steamer for 40 min and allowed to cool for 20 min and rinsed in PBS. After treated with 0.1% Triton X-100 in PBS for 5 min, tissue sections were incubated with 5% normal donkey serum in PBS for 1 h followed by incubation of primary antibodies: rat anti-PyVT (Santa Cruz Biotechnology) and rabbit anti-ErbB2 (Thermo Fisher Scientific). Tissue sections were then washed three times in PBS for 5 min each and incubated with the appropriate secondary antibodies of either Alexa Fluor 594-conjugated goat anti-rat or Alexa Fluor 594-conjugated goat anti-rabbit (all antibodies from Thermo Fisher Scientific) for the triple fluorescence studies or an alkaline phosphatase-conjugated goat anti-rat (Sigma-Aldrich Chemicals, St. Louis, MO) or alkaline phosphatase-conjugated goat anti-rabbit (Abcam) for the immunocytochemical studies. The color was developed with A Fast Red precipitating chromogenic substrate system. For these immunocytochemical studies, the slides were counterstained with hematoxylin. For the fluorescence studies, the sections were finally mounted with Vectorshield mounting medium with DAPI (Vector Laboratories) and viewed with an Olympus Fluoview-1000 confocal scanning system. For the immunocytochemical studies, the slides were viewed with an Olympus microscope with attached digital camera.

#### Quantitative digital image analysis

Multiple 2-mm tissue cores of tumor from each paraffin-embedded donor block were arrayed into a new recipient paraffin block.

Our specific TMA algorithms took a whole virtual TMA and carried out virtual alignment and core indexing. In this manner, a virtual TMA could be created consisting of perfectly oriented horizontal and vertical linear arrays of tissue cores. The process of scanning each TMA slide into a virtual slide took approximately 30 min. Subsequent virtual alignment, image processing, and the application of the epithelial recognition algorithms (ERAs) and specific recognition algorithms (SRAs) took an additional 30 min/TMA.

Image acquisition by either the Aperio ScanScope T2 System (Aperio, Vista, CA) or the iSCAN System (BioImagene, Inc., Cupertino, CA) produced equivalent results with uniformly produced sharp images with high contrast. For approximately 5% of the acquired images, the image quality was below the standard where the algorithms were interpretative. These images had to be discarded. For approximately 10% of the images, mask removal and contrast enhancement improved image quality.

ERAs applied to each TMA core were successful in recognizing epithelium, filtering out stroma and determining its epithelial percentage and therefore its cancer cell density. Specific immunoreactivity was then analyzed by the application of the appropriate SRAs which included both cytoplasmic and membrane recognition algorithms. The ability of the algorithms to recognize the cellular compartments of the cancer cell, detect the appropriate immunoreactivity which was present and quantitate it on both ordinal as well as continuous scales has been demonstrated previously [27–29]. The algorithm-based determination of immunoreactivity was the same every time the algorithm was run and therefore showed no interobserver, intraobserver, or fatigue variability.

### Statistical analysis

All experiments were performed with a minimum of three replicates and representative results depicted. Declarations of differences imply differences of statistical significance. Significance was assessed by the Student’s *t* test.

## Supplementary Information

**Additional file 1.**

**Additional file 2.**

## Data Availability

All iPSC clones used in the study are available to scientists upon request. No data sets, per se, were generated in the study.

## Abbreviations

iPSCs: Induced pluripotent stem cells
MMTV: Mouse mammary tumor virus
PyVT: Polyomavirus middle T antigen
SSEA-1: Mouse-specific stage-specific embryonic antigen-1
ERAs: Epithelial recognition algorithms
SRAs: Specific recognition algorithms
GFP: Green fluorescence protein
DCIS: Ductal carcinoma in situ
TMA: Tissue microarray
WAP: Whey acidic protein
FACS: Fluorescence-activated cell sorting
TFs: Transcription factors
PI-MECs: Parity-induced mouse mammary epithelial cells
mES: Murine embryonic stem cells
