# Correction: Use of constitutive and inducible oncogene-containing iPSCs as surrogates for transgenic mice to study breast oncogenesis

**DOI:** 10.1186/s13287-025-04492-2

**Published:** 2025-07-10

**Authors:** Christine Nguyen, Julie P. T. Nguyen, Arnav P. Modi, Ihsaan Ahmad, Sarah C. Petrova, Stuart D. Jr Ferrell, Sabrina R. Wilhelm, Yin Ye, Dorthe Schaue, Sanford H. Barsky

**Affiliations:** 1 Cancer Center and Institute for Personalized Medicine, California University of Science and Medicine (CUSM), 1501 Violet Street, Colton, CA 92324 USA; 2 Department of Radiation Oncology, David Geffen School of Medicine, University of California, Los Angeles, CA 90095-1714 USA

**Correction: Stem Cell Research & Therapy (2021) 12:301**

10.1186/s13287-021-02285-x

The authors note the following corrected text for the legend of Fig. 6 which has been slightly reworded for greater accuracy:

Fig 6. Trifluorescence and colorimetric studies of stages of oncogenesis on select iPSC clones. Triple fluorescence studies on PyVT-iPSC clone derived extirpated tumors displayed DAPI blue nuclear autofluorescence (a), GFP green autofluorescence (b), Alexa Fluor® 594 red immunofluorescence using goat anti-rat added to rat monoclonal to PyVT antigen (c) and its merged overlay (d). Expression of PyVT is detected not only within the areas of invasive carcinoma (yellow arrow) but also within the normal breast ducts (red arrow) and breast ducts containing ductal carcinoma in situ (DCIS) (green arrow) whereas murine angiogenesis (dark areas) did not exhibit any GFP autofluorescence or immunofluorescence. Colorimetric immunocytochemistry studies utilizing A Fast Red precipitating chromogenic substrate system coupled with alkaline phosphatase conjugated goat anti rat and rat anti-PyVT revealed red chromogenicity not only within the invasive carcinoma but also within adjacent normal ducts (red arrow) (e). The iPSC clones derived from the tri-transgenic MMTV-PyVT/MMTV-cre/Rosa26LoxP, when injected orthotopically gave rise to normal ductal system development (f) and stages of breast cancer progression (f), both identified colorimetrically. The iPSC clones were capable of behaving orthotopically no different than that which was observed in their bi/tritransgenic parents (g,h), the latter two sub-panels adapted from 10.1158/0008-5472.CAN-07-2148 with permission from AACR.

