# Supplementary figures and images for: Use of constitutive and inducible oncogene-containing iPSCs as surrogates for transgenic mice to study breast oncogenesis
Source: Stem Cell Res Ther. 2021 May 27;12:301. doi: 10.1186/s13287-021-02285-x (PMC8162012; doi:10.1186/s13287-021-02285-x)

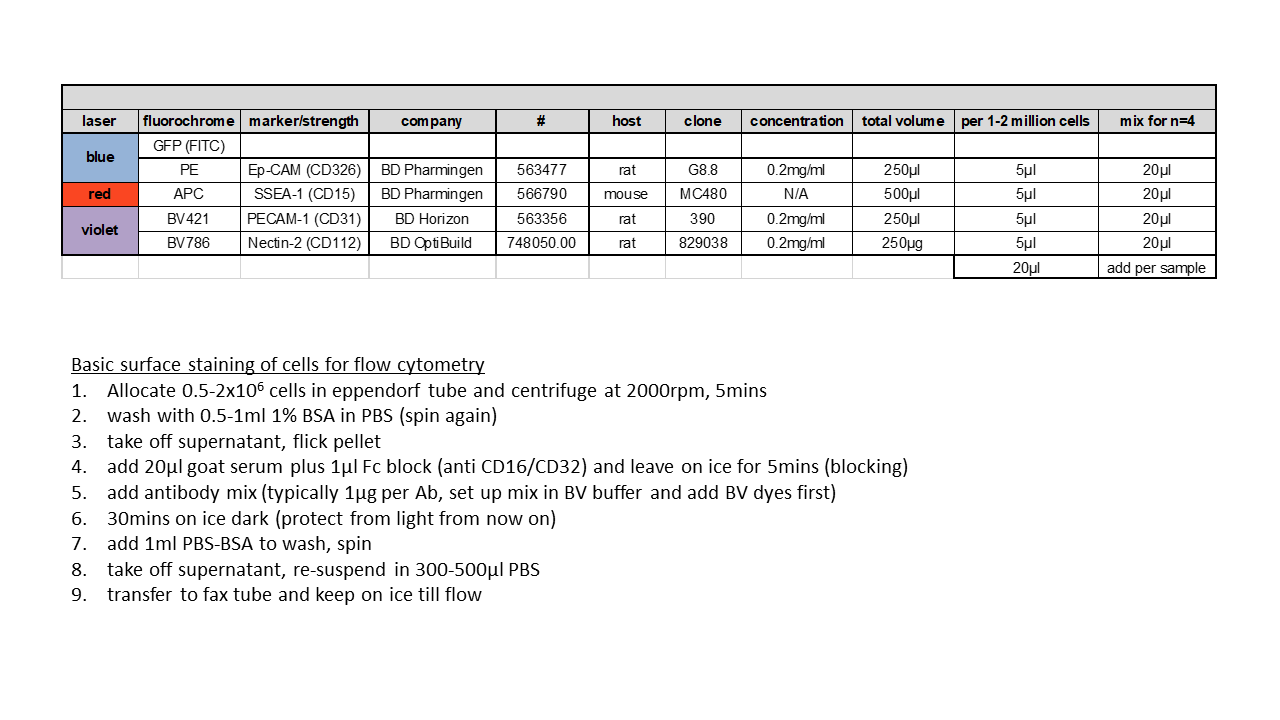

Supplement: Supplementary file 1 — Additional file 1. [file 13287_2021_2285_MOESM1_ESM.zip › Supplement 1a.TIF]

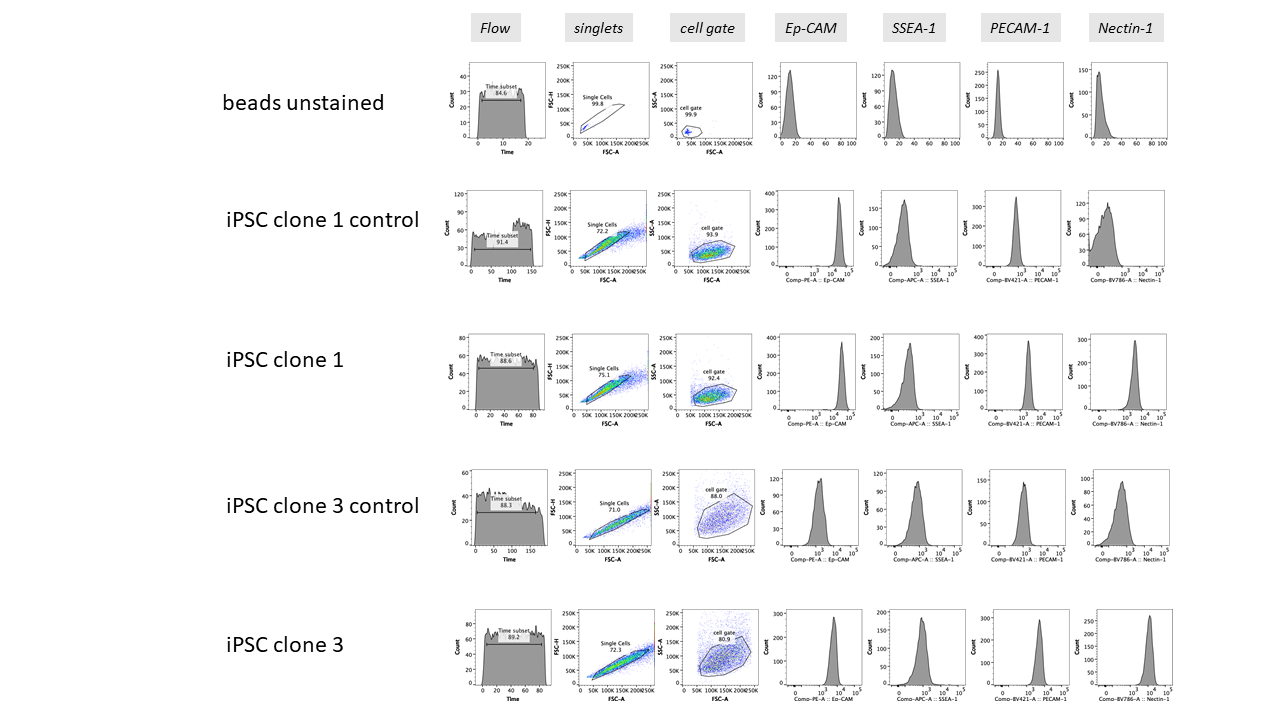

Supplement: Supplementary file 1 — Additional file 1. [file 13287_2021_2285_MOESM1_ESM.zip › Supplement 1b.TIF]

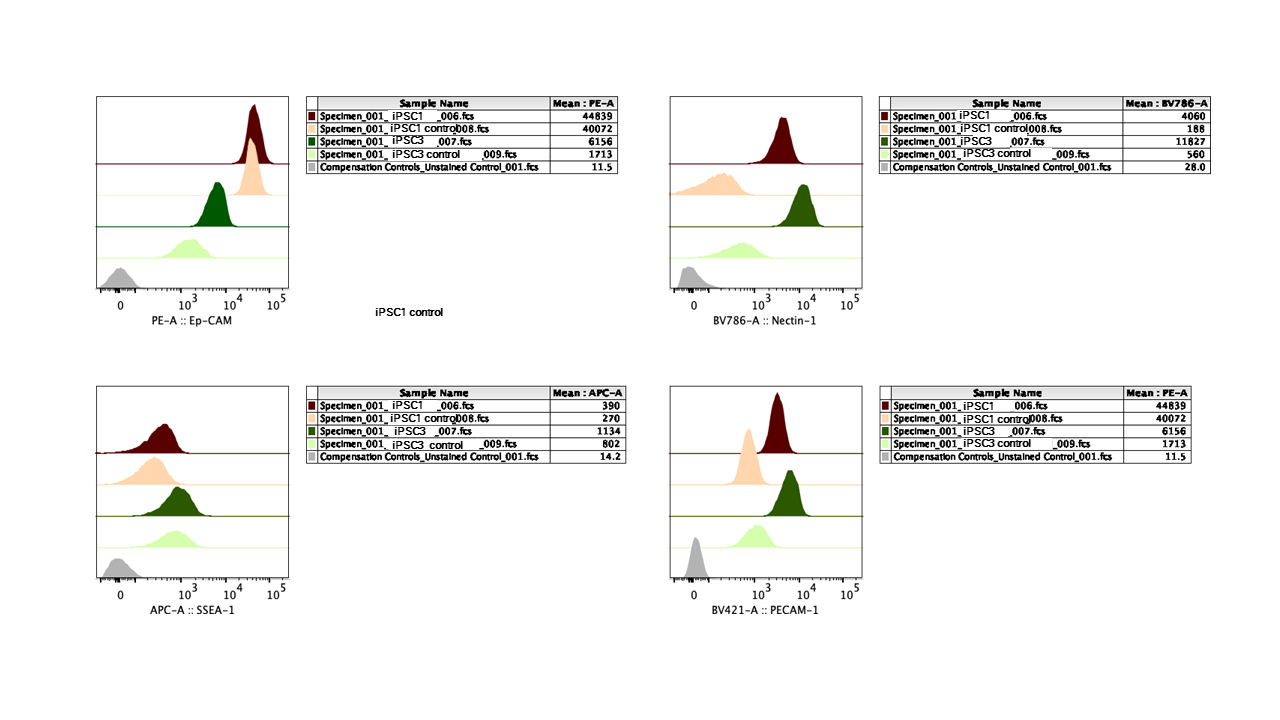

Supplement: Supplementary file 1 — Additional file 1. [file 13287_2021_2285_MOESM1_ESM.zip › Supplement 1c.TIF]

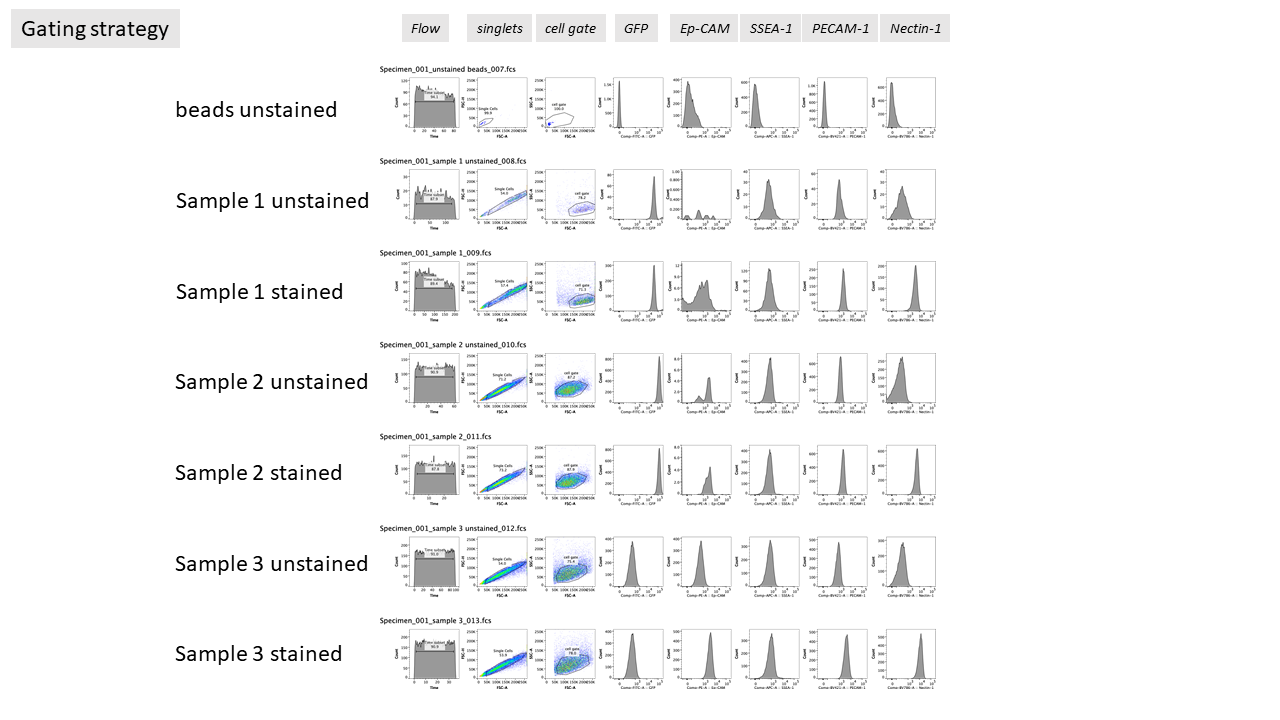

Supplement: Supplementary file 1 — Additional file 1. [file 13287_2021_2285_MOESM1_ESM.zip › Supplement 1d.TIF]

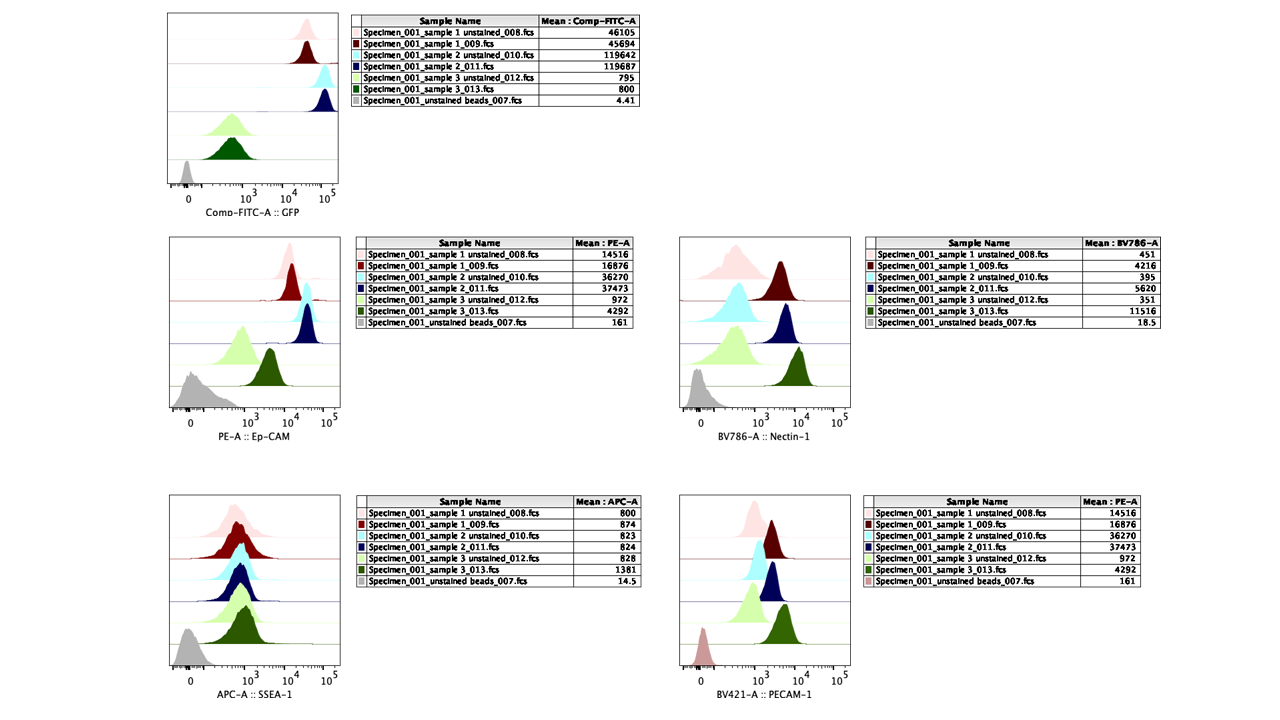

Supplement: Supplementary file 1 — Additional file 1. [file 13287_2021_2285_MOESM1_ESM.zip › Supplement 1e.TIF]
